# Genome-wide association study reveals putative role of gga-miR-15a in controlling feed conversion ratio in layer chickens

**DOI:** 10.1186/s12864-017-4092-9

**Published:** 2017-09-06

**Authors:** Jingwei Yuan, Sirui Chen, Fengying Shi, Guiqin Wu, Aiqiao Liu, Ning Yang, Congjiao Sun

**Affiliations:** 1 National Engineering Laboratory for Animal Breeding and MOA Key Laboratory of Animal Genetics and Breeding, College of Animal Science and Technology, China Agricultural University, Beijing, China; 2 Beijing Engineering Research Center of Layer, Beijing, China

**Keywords:** Feed efficiency, Genome-wide association study, Gga-miR-15a, Late laying period, Chickens

## Abstract

**Background:**

Efficient use of feed resources for farm animals is a critical concern in animal husbandry. Numerous genetic and nutritional studies have been conducted to investigate feed efficiency during the regular laying cycle of chickens. However, by prolonging the laying period of layers, the performance of feed utilization in the late-laying period becomes increasingly important. In the present study, we measured daily feed intake (FI), residual feed intake (RFI) and feed conversion ratio (FCR) of 808 hens during 81–82 weeks of age to evaluate genetic properties and then used a genome-wide association study (GWAS) to reveal the genetic determinants.

**Results:**

The heritability estimates for the investigated traits were medium and between 0.15 and 0.28 in both pedigree- and genomic-based estimates, whereas the genetic correlations among these traits were high and ranged from 0.49 to 0.90. Three genome-wide significant SNPs located on chromosome 1 (GGA1) were detected for FCR. Linkage disequilibrium (LD) and conditional GWA analysis indicated that these 3 SNPs were highly correlated with one another, located at 13.55–45.16 Kb upstream of gga-miR-15a. Results of quantitative real-time polymerase chain reaction (qRT-PCR) analysis in liver tissue showed that the expression of gga-miR-15a was significantly higher in the high FCR birds than that in the medium or low FCR birds. Bioinformatics analysis further revealed that gga-mir-15a could act on many target genes, such as forkhead box O1 (*FOXO1*) that is involved in the insulin-signaling pathway, which influences nutrient metabolism in many organisms. Additionally, some suggestively significant variants, located on GGA3 and GGA9, were identified to associate with FI and RFI.

**Conclusions:**

This GWA analysis was conducted on feed intake and efficiency traits for chickens and was innovative for application in the late laying period. Our findings can be used as a reference in the genomic breeding programs for increasing the efficiency performance of old hens and to improve our understanding of the molecular determinants for feed efficiency.

**Electronic supplementary material:**

The online version of this article (10.1186/s12864-017-4092-9) contains supplementary material, which is available to authorized users.

## Background

People are always paying attention to animal feed efficiency because of the large effect on farm profitability. For the poultry industry, feed efficiency represents its competitive position against other animal protein sources, and to food economists, efficiency places less demand on global feed resources [1]. The advances in optimizing diet formulations have significantly improved the feed efficiency for layers in the past decades. However, with increasing feed costs, further improvement by genetics and breeding strategies is a particularly important aspect. By integrating statistical genetics, molecular biology and sequencing technology in numerous studies, the genetic determinants for many economic traits of farm animals have been revealed, such as the blue eggshell in chicken [2], glycogen content of skeletal muscle in pig [3], and pleiotropic polymorphisms for stature, fatness and reproduction traits in beef [4]. Poultry geneticists have focused on elucidating the genetic mechanisms that determine feed efficiency, such as identifying quantitative trait loci (QTLs) and genomic variants in chicken [5], waterfowl [6], turkey [7], and quail [8], among others. However, most of the screened loci that putatively influence feed efficiency are breed-, age-, or breeding area-specific (http://www.animalgenome.org/cgi-bin/QTLdb/GG/index). The previous findings indicate that feed efficiency as a variably quantitative trait requires a more accurate and comprehensive strategy to reveal the genetic factors for birds under several conditions.

In the egg-type chicken industry, a trend has developed to prolong the laying cycle, which is related to animal welfare, the ecological footprint of animal production and the use of natural resources [9]. However, the decline of performance for old hens represents a substantial challenge for this development pattern. Accordingly, to prolong persistent bird performance, feed efficiency must be addressed. To achieve the above goals, genetic determinants for feed efficiency of old hens require investigation. In the current study, feed efficiency traits were measured for layers 81–82 weeks of age, and then the GWAS method with a molecular validation strategy was used to detect the genetic variants and candidate genes that were related to feed efficiency.

## Results

### Phenotypic descriptions and genetic properties

The descriptive statistics of daily feed intake (FI), residual feed intake (RFI), feed conversion ratio (FCR), body weight (MBW) and daily egg mass (EM) are presented in Table 1 for 808 qualified hens. Chickens consumed an average of 122 g of feed and produced ~50 g of egg mass per day in the laying period of 81–82 weeks of age. The minimum and maximum values of RFI were −41.65 g/d and 43.69 g/d, respectively. The coefficient of variation (CV) of FCR (20.18%) was higher than that of FI (11%). The raw data of RFI were normally distributed, and the data of FI and FCR fitted a normal distribution after Johnson transformation.

**Table 1:** Descriptive statistics of feed efficiency and related traits^a^

Traits^b^	Mean	SD	CV(%)	Min	Max
FI (g/d)	121.87	13.41	11.00	70.70	165.06
RFI (g/d)	0	12.47	–	−41.65	43.69
FCR (g:g)	2.51	0.51	20.18	1.58	4.73
EM (g/d)	48.55	8.74	17.48	15.57	71.64
MBW (g)	2236.9	182.9	8.16	1638.0	2936.0

^a^n = 808

^b^FI, RFI, FCR, EM and MBW represent daily feed intake, residual feed intake and feed conversion ratio, daily egg mass and mean body weight, respectively

Estimates of heritability and genetic correlations among these traits are listed in Table 2. Pedigree-based heritability estimates for FI (0.18 ± 0.07) and RFI (0.20 ± 0.07) were lower than that for FCR (0.28 ± 0.09). Compared with pedigree-based estimates, the SNP-based heritability estimates were lower for FI (0.15 ± 0.05), RFI (0.17 ± 0.05) and FCR (0.21 ± 0.05). Regarding the genetic correlations, SNP-based estimates were a little different from pedigree-based estimates. The highest correlation was found between FI and RFI (0.90 ± 0.05 and 0.86 ± 0.06 for pedigree- and SNP-based estimates, respectively). By contrast, the lowest correlations for pedigree- and SNP-based estimates were found between FI and FCR at 0.49 and 0.39 with substantial standard errors of 0.22 and 0.20, respectively.

**Table 2:** Genetic parameters for daily feed intake, residual feed intake and feed conversion ratio^a^

Traits^b^	FI	RFI	FCR
FI	**0.15 (0.05)**	0.86 (0.06)	0.39 (0.20)
	***0.18 (0.07)***		
RFI	*0.90 (0.05)*	**0.17 (0.05)**	0.71 (0.13)
		***0.20 (0.07)***	
FCR	*0.49 (0.22)*	*0.71 (0.15)*	**0.21 (0.05)**
			***0.28 (0.09)***

^a^Heritability is given on diagonal (italic bold is pedigree-based heritability and bold is SNP-based), pedigree-based genetic correlations below diagonal and SNP-based genetic correlations above diagonal. Standard errors of estimates are in parentheses

^b^FI: daily feed intake, RFI: residual feed intake, FCR: feed conversion ratio

### Genome-wide association study

**Fig. 1:**
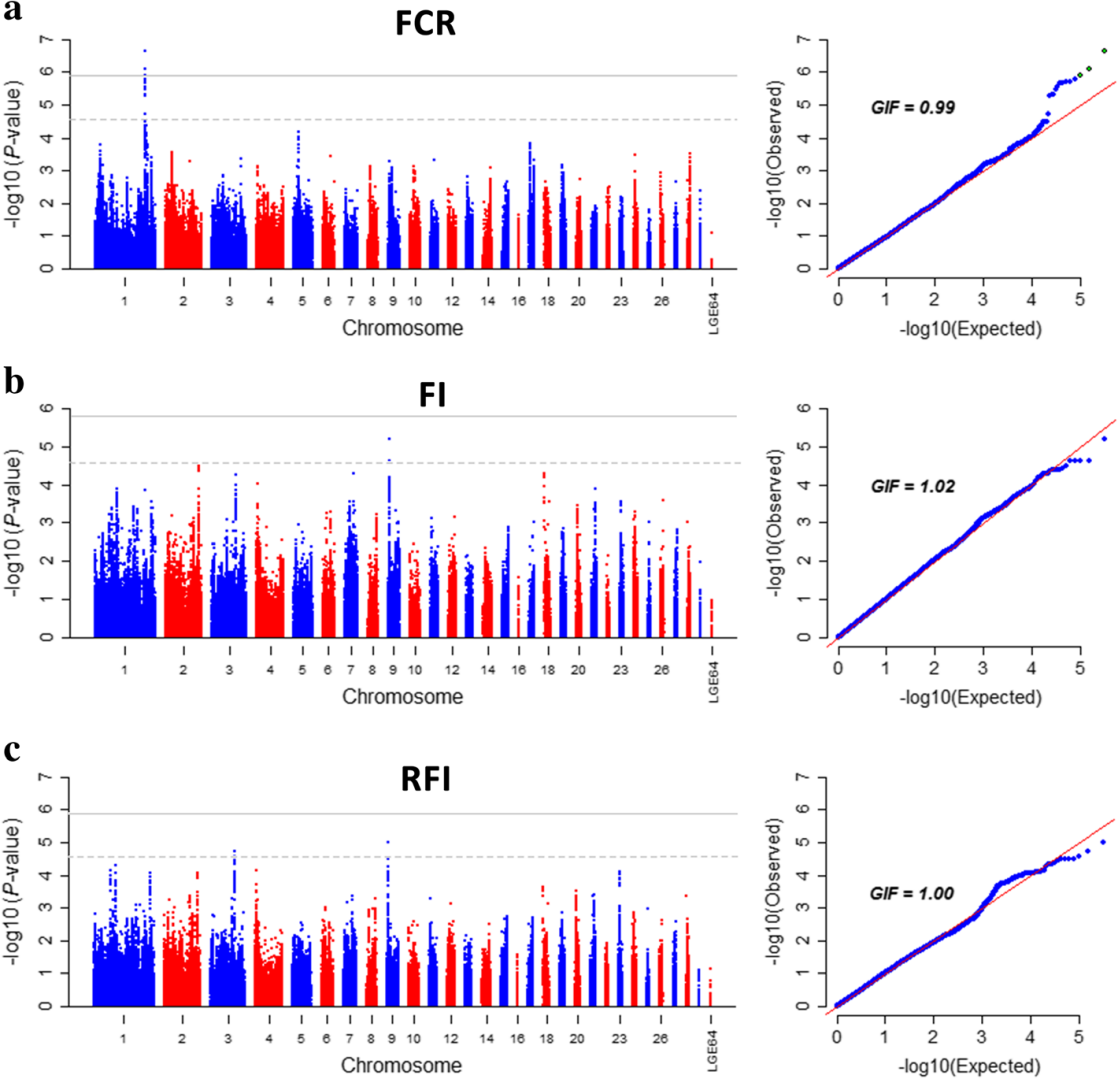
Manhattan and Q-Q plot of genome wide association study for feed intake and efficiency traits. Each dot represents a SNP in the dataset. The horizontal gray line and gray dashed line indicate the genome-wise significance threshold (*P* value = 1.29e-6) and genome-wise suggestive significance threshold (*P* value = 2.58e-5), respectively. FI, RFI and FCR denote daily feed intake, residual feed intake and feed conversion ratio, respectively. GIF represents genomic inflation factor. **a**) Plot for feed conversion ratio, **b**) Plot for daily feed intake, **c**) Plot for residual feed intake

**Fig. 2:**
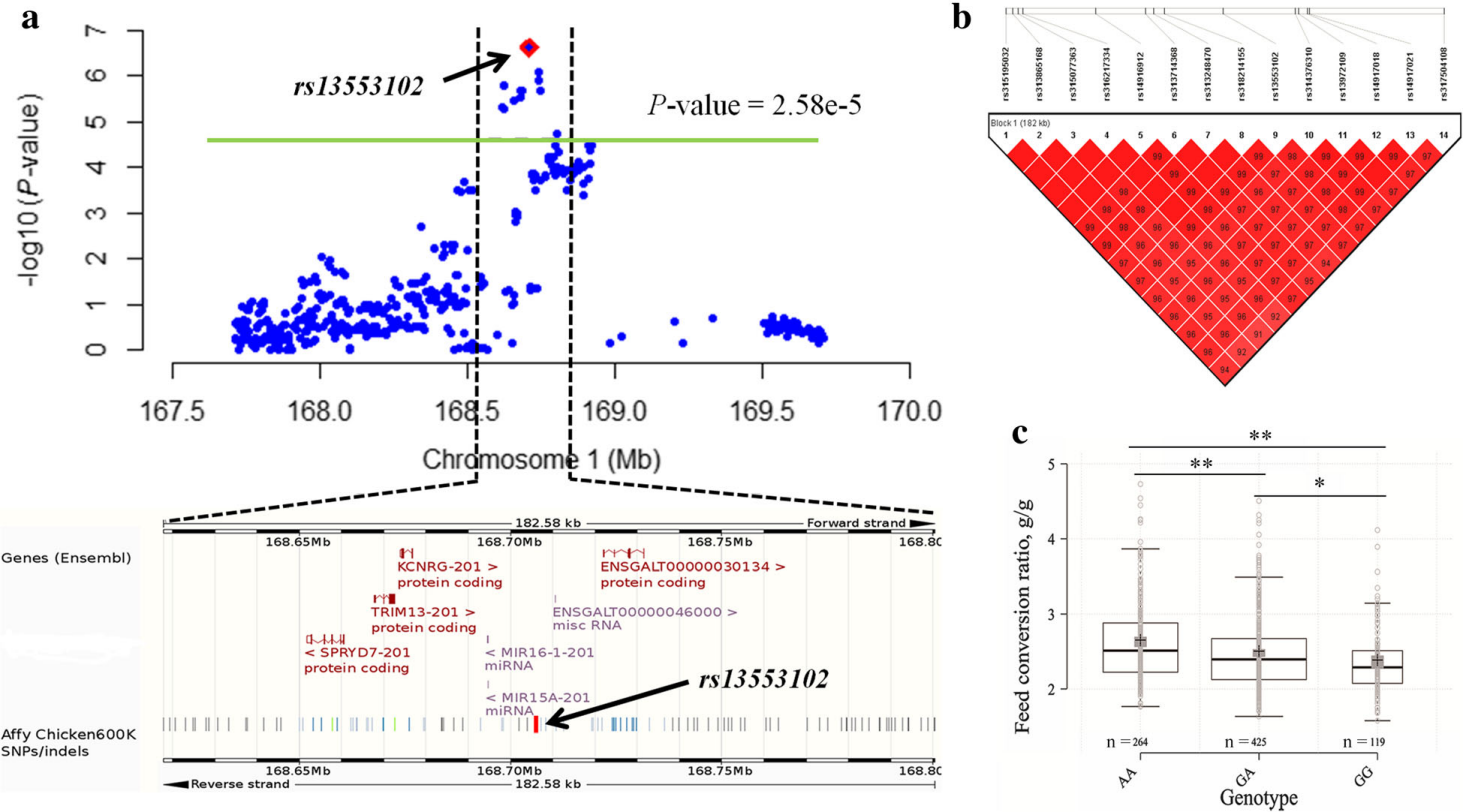
Association results of candidate region on chromosome 1 (GGA1) for feed conversion ratio (FCR). **a** Location of the loci associated with FCR on GGA1. The graph plots genomic position (x axis) against its significance expressed as -log10 *P* value (y axis). Genomic position of associated SNPs reaching suggestive significance (*P*-value = 2.58e-5) indicated by a green horizon line span 182.58 kb. The SNP *rs13553102* is red highlighted. The annotated candidate genes and SNP displayed below the graph downloaded from Ensembl database. **b** Linkage disequilibrium (LD) plot for the 14 SNPs reaching suggestive significance in the candidate region on GGA1. **c** Genotype effect plot of the SNP *rs13553102*. **(*P* < 0.01) and *(*P* < 0.05) indicate significant differences among groups (*n* = 264, 425 and 119 for AA, GA and GG, respectively)

The Manhattan and quantile-quantile (Q-Q) plots for FCR, FI and RFI are shown in Fig. 1. Genome-wide association analysis revealed 3 genome-wide significant SNPs (Table 3) and 11 suggestively significant SNPs (Additional file 1: Table S1) associated with FCR (Fig. 2a). These SNPs were in high linkage disequilibrium (Fig. 2b) and located in a region that ranged from 168.62 to 168.80 Mb on GGA1. Together, these 14 SNPs explained 2.30% of the phenotypic variance of FCR. Gga-miR-15a (*MIR15A*) was the only gene near these 3 genome-wide significant loci. Several genes also harbored or were near the 11 suggestively significant SNPs, including gga-miR-16a-1 (*MIR16–1*), deleted in lymphocytic leukemia 2 (*DELU2*), SPRY domain containing 7 (*SPRYD7*), potassium channel regulator (*KCNRG*) and tripartite motif containing (*TRIM13*). To further test the possible secondary association signals at the locus, conditional GWA analysis was conducted with the top associated SNP *rs13553102* as a covariate*.* All SNPs were hidden below the suggestively significant line (Additional file 2: Figure S1) after conditional GWA analysis, which suggested that SNP *rs13553102* was the most reliable signal in this region. The SNP was located at 13.55 Kb upstream of *MIR15A* with a MAF of 0.41. The substitution of variant A to G for *rs13553102* led to a significant decrease in FCR value (Fig. 2c). The genomic inflation factor (GIF) was 0.99 for FCR and indicated that the population stratification was well corrected in the analysis.

**Table 3:** The information for SNPs associated with feed intake and efficiency traits

Traits^a^	SNP	GGA^b^	Position	*P*-value^c^	MAF^d^	β^e^	Candidate/nearest gene	Location (kb)^f^
FCR	rs13553102	1	168,708,318	2.35e-7*	0.41 (A/G)	−0.29	MIR15A	U 13.57
	rs314376310	1	168,738,343	7.93e-7*	0.51 (G/C)	−0.29	MIR15A	U 43.57
	rs13972109	1	168,739,928	1.27e-6*	0.50 (T/C)	−0.28	MIR15A	U 45.16
FI	rs313839239	9	4,521,384	6.21e-6	0.06 (T/C)	0.56	FARP2	Intron 1
	rs313750381	9	4,358,988	2.47e-5	0.04 (A/G)	0.59	KIF1A	Intron 22
	rs314936159	9	4,371,299	2.47e-5	0.04 (A/G)	0.59	KIF1A	U 0.59
	rs313292633	9	4,397,583	2.47e-5	0.04 (T/C)	0.59	SNED1	Exon 11
	rs312606176	9	4,402,911	2.47e-5	0.04 (G/A)	0.59	SNED1	Intron 22
RFI	rs314723494	3	75,533,793	1.94e-5	0.33 (T/C)	3.17	CNR1	U 46.60
	rs313839239	9	4,521,384	1.01e-5	0.06 (T/C)	6.74	FARP2	Intron 1

^a^FCR, FI and RFI represent daily feed intake, residual feed intake and feed conversion, respectively

^b^Chicken chromosome

^c^* Indicates that the SNP *P* value reaches a genome-wise significance

^d^Allele frequency of the first listed marker

^e^Effect of allele substitution

^f^U indicates that the SNP is upstream of a gene 5′-UTR

**Fig. 3:**
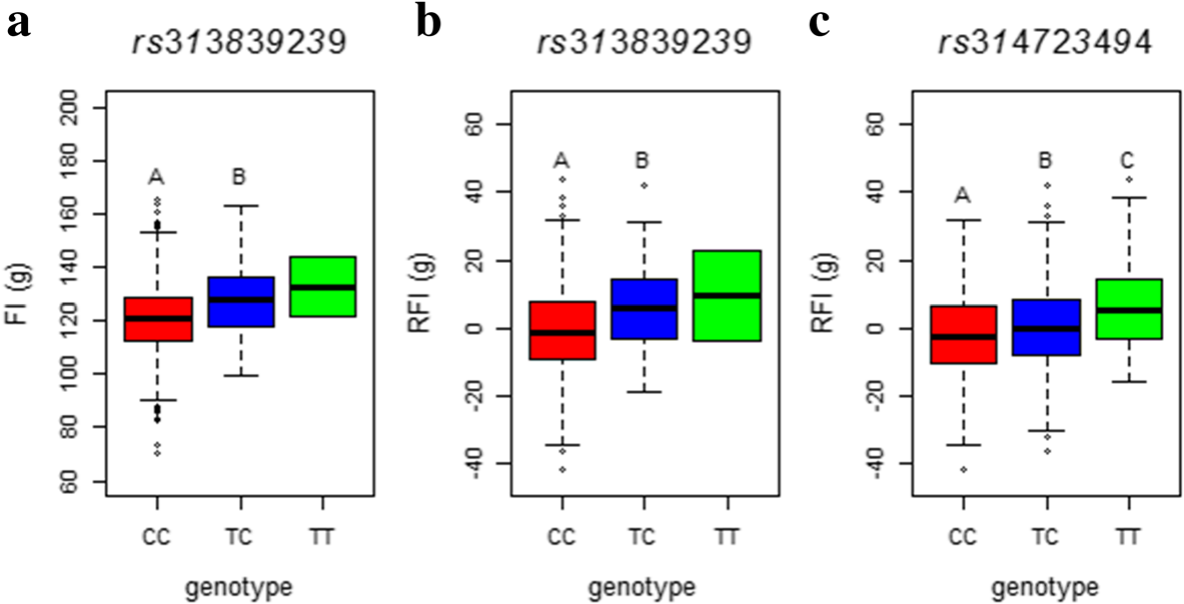
Boxplot of SNP effect for daily feed intake and residual feed intake. **a** The effect of *rs313839239* on daily feed intake. **b** The effect of *rs313839239* on residual feed intake. **c** The effect of *rs314723494* on daily feed intake. Boxes with different letters are significantly (*P* < 0.05) different from each other. FI and RFI denote daily feed intake and residual feed intake, respectively

For FI and RFI, we didn’t find any genome-wide significant hit. However, a handful of secondary important SNPs were identified at a suggestively significant level. Five suggestively significant SNPs with a low minor allele frequency (MAF < 0.1) were detected for FI on chromosome 9 (GGA9) spanning from 4.36 to 4.52 Mb. All SNPs had a positive effect on FI. Linkage disequilibrium (LD) analysis showed that the 5 SNPs were in a high linkage phase (Additional file 3: Figure S2), suggesting that a potential QTL affecting FI might be harbored in this region. The candidate genes in this region included *FARP2* (FERM, RhoGEF and pleckstrin domain protein 2), *KIF1A* (kinesin family member 1A) and *SNED1* (sushi, nidogen and EGF-like domains 1). Compared with the FI, only two SNPs, *rs314723494* and *rs313839239*, with genome-wide suggestive significance were detected for RFI. The two SNPs were located on GGA3 and GGA9. The identical SNP *rs313839239* identified for FI and RFI might support the high genetic correlation between FI and RFI (Table 2). SNP *rs313839239* had a low frequency of minor allele T (MAF = 0.06) in the current population. However, the substitution of variant C to T caused a significant difference for both FI (Fig. 3a) and RFI (Fig. 3b) values. Another SNP *rs314723494* with a positive effect on the RFI was located in the 46.6 kb upstream of cannabinoid receptor 1 (*CNR1*). Chickens with the CC genotype of *rs314723494* were more efficient with a − 1.88 g/d RFI than TC and TT genotypes with 0.27 and 6.53 g/d RFI, respectively (Fig. 3c). Moreover, the GIF was 1.02 and 1.00 for FI and RFI, respectively, indicating that the association analyses were scarcely affected by the population stratification.

### Expression of gga-miR-15a in liver tissue

**Fig. 4:**
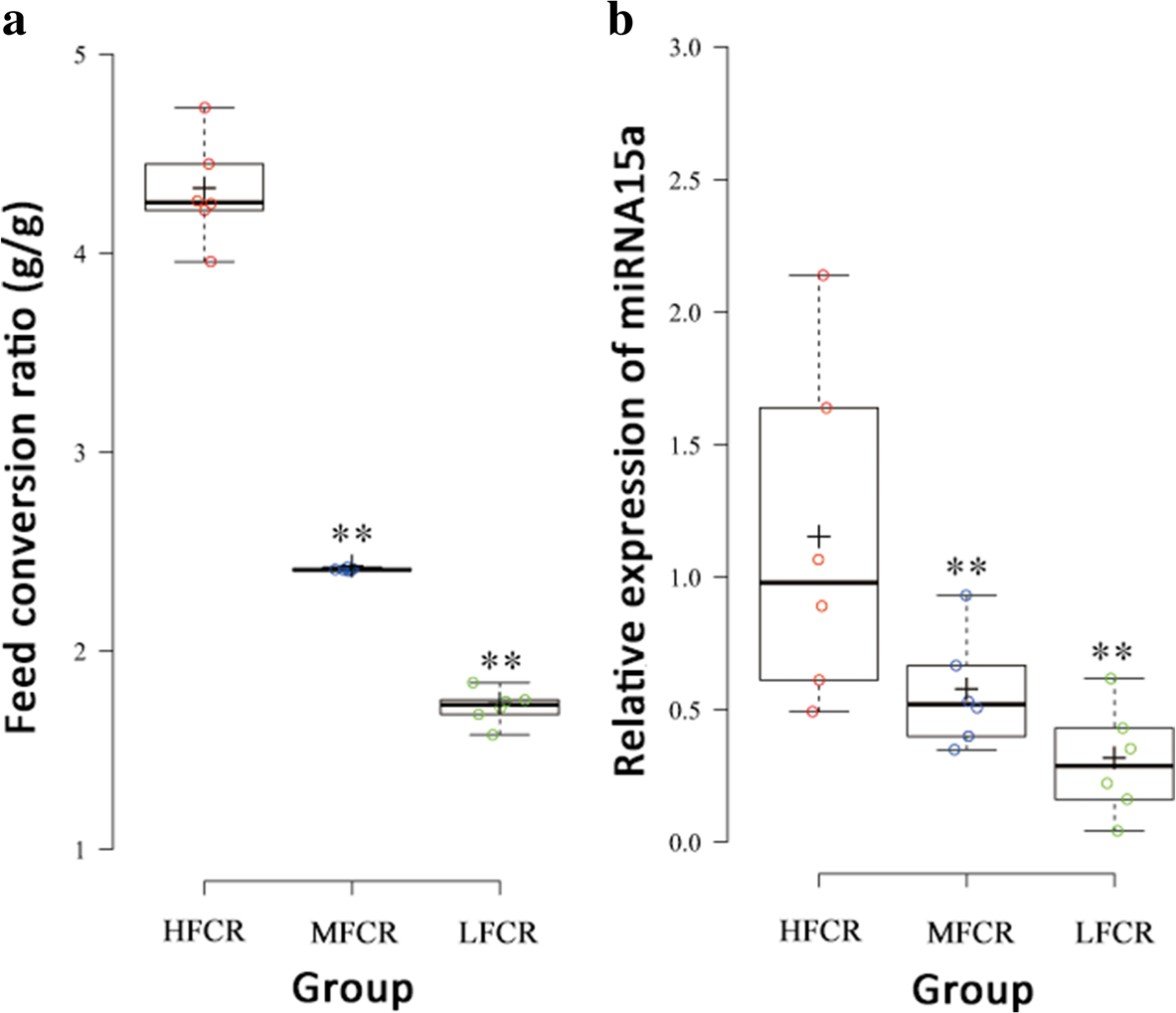
Expression of gga-mir-15a for hens with high, medium and low feed conversion ratio. **a** The phenotype of feed conversion ratio for hens selected from high, medium and low feed conversion ratio group. **b** Expression of gga-mir-15a for the selected hens. Gene expression is presented relative to 5 s RNA expression and normalized to a calibrator. **P < 0.01. Six birds per group were available for the analysis

According to the association analysis of FCR, all genome-wide significant SNPs were near gga-miR-15a. We considered gga-miR-15a a promising candidate that might be associated with feed efficiency. Therefore, based on the FCR values (Fig. 4a) only, we selected six birds from high (HFCR), medium (MFCR) and low FCR (LFCR) groups and then extracted the total RNA of their liver tissue. The cDNA was used to run quantitative real-time PCR (qRT-PCR) for gga-miR-15a. We found that the relative expression of gga-miR-15a was significantly higher in the HFCR group than that in MFCR and LFCR groups (Fig. 4b), suggesting that the gga-miR-15a should be a promising candidate gene for feed efficiency.

### Target gene prediction for gga-miR-15a

**Fig. 5:**
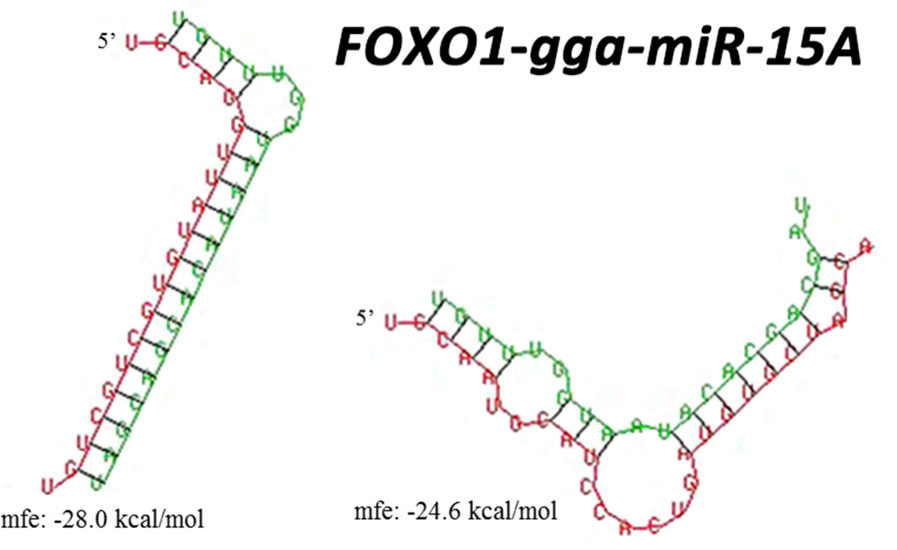
Molecular interactions between gga-miR-15a and 3 prime untranslated regions (3′-UTR) of *FOXO1*. Red letters indicate the 3′-UTR sequences of the target genes. Green letters indicate the matured sequences of gga-miR-15a. MFE represents minimal free energy

To investigate the possible mechanism for gga-miR-15a to influence feed efficiency, further bioinformatics analysis was performed. TargetScan and miRDB software was used for target gene prediction for gga-miR-15a, and a total of 196 and 363 (target score ≥ 80) genes were predicted using these two tools, respectively (Additional file 1: Tables S2 and S3). These target genes were pooled to perform pathway analysis on the DAVID platform after which target genes were significantly (*P* < 0.05) enriched to 9 biological pathways (Table 4) against the database of the Kyoto Encyclopedia of Genes and Genomes (KEGG). After Benjamini *p*-value correction, only the insulin-signaling pathway (gga-04910) was significantly enriched, with 12 target genes involved. Interaction analysis was then performed on these 12 genes one by one using RNA hybrids. Minor free energy (MFE) was selected as an indicator to identify the reliable bind between microRNA and target mRNA. A total of 9 interactions were found between gga-miR-15a and three target genes with an MFE less than −20. These 3 genes were forkhead box O1 (*FOXO1*), 3-phosphoinositide dependent protein kinase 1 (*PDPK1*) and protein kinase cAMP-dependent type II regulatory subunit alpha (*PRKAR2A*) (Additional file 4: Figure S3), and the lowest MFE was found between gga-miR-15a and *FOXO1*, which showed two binding sites with −28 and −24.6 kcal/mol MFE (Fig. 5).

**Table 4:** Significant KEGG pathways for target genes of gga-mir-15a

KEGG^a^ pathway	Count	%	Involved genes	P value	Benjamini^b^
Insulin signaling pathway	12	2.48	PDPK1, PRKAR2A, CRKL, PHKA1, SOS2, FOXO1, RAF1, MAPK8, IRS1, INSR, PIK3R1, AKT3	1.64E-03	4.90E-02
mTOR signaling pathway	8	1.65	PDPK1, RPS6KA3, ULK1, CAB39, RICTOR, IRS1, PIK3R1, AKT3	1.18E-03	0.05
FoxO signaling pathway	13	2.69	USP7, SGK1, FOXO1, RAF1, IRS1, CCND1, PDPK1, CDKN2B, SOS2, MAPK8, INSR, AKT3, PIK3R1	6.79E-04	0.06
Oocyte meiosis	9	1.86	CCNE1, RPS6KA3, YWHAH, CPEB2, CPEB3, BTRC, PPP2R5C, YWHAQ, ITPR2	0.01	0.20
MAPK signaling pathway	15	3.10	TAOK1, NF1, PPM1A, PTPRR, RAF1, RPS6KA3, CRKL, MAP3K4, MAP3K2, ELK4, SOS2, MAPK8, RAPGEF2, NFATC3, AKT3	0.01	0.21
Wnt signaling pathway	10	2.07	TBL1XR1, CCND1, NKD1, BTRC, LRP6, MAPK8, SIAH1, FZD3, NFATC3, WNT7A	0.02	0.26
Insulin resistance	8	1.65	PDPK1, RPS6KA3, FOXO1, MAPK8, IRS1, INSR, PIK3R1, AKT3	0.04	0.40
Progesterone-mediated oocyte maturation	7	1.45	RPS6KA3, CPEB2, CPEB3, RAF1, MAPK8, PIK3R1, AKT3	0.04	0.42

^a^Kyoto Encyclopedia of Genes and Genomes

^b^Benjamini-Hochberg false discovery rate ≤ 0.10

## Discussion

Genetic analysis was conducted in layer chickens affiliated with a nucleus breeding population. Birds were measured for feed intake and efficiency traits at an age greater than 80 weeks. To our knowledge, this study is the first genetic analysis for feed efficiency traits in the late laying period of chickens. Compared with the previous GWAS in the F_2_ resource population [5], the diversity of SNPs was decreased by quality control of minor allele frequency in the current study. This decrease might be due to the long-term selection scheme performed in the nucleus breeding population, which led to many homozygous alleles occurring in the genome. Moreover, the few and different associated hits detected in the current study might be explained by the difference of laying period and population structure [10].

The pedigree-based heritability estimates of FI and RFI presented here were substantially lower than those in the F_2_ population, which were evaluated at the age of 40 and 60 weeks, whereas the estimate of FCR was much higher in the current population. This suggested that different genetic backgrounds could affect the estimates of heritability, as the present study used population that had been selected for egg production for many generations. The high genetic correlation between FI and RFI but not FCR is consistent with our previous findings [11], suggesting that the genetic foundation of RFI is more closely related with that of FI for layer chickens. Additionally, the SNP-based heritability estimates were smaller than pedigree-based estimates, which were likely caused by the “missing heritability” [12] that cannot be explained by common SNPs on the 600 K SNP array.

A genomic region of 31 kb on chromosome 1 (GGA1) that harbored 3 genome-wide significant SNPs was detected associated with the feed conversion ratio (FCR). Additionally, gga-mir-15a (*MIR15A*) was in this region and close to the three significant SNPs. Given that FCR is related to energy homeostasis and egg production, we constructed cDNA from liver tissue, which is vital to glucose, glucagon, lipid and protein metabolism in chickens [13–15], to conduct a gene expression experiment, i.e., qRT-PCR. The expression of *MIR15A* was significantly lower in the medium and low FCR birds, which suggested that *MIR15A* was a promising candidate gene involved in the regulation of FCR. MicroRNAs are small non-coding RNAs that have been highly conserved during evolution and have been implicated in multiple molecular interactions [16]. According to the genome of vertebrates, *MIR15A,* accompanied by *MIR16–1* and *DELU2* nearby, forms a *DLEU2/miR-15a/16–1* cluster to affect chronic lymphocytic leukemia in cancer research [17, 18]. As an important independent regulatory molecule, microRNA-15a is involved in the regulation of cell apoptosis and proliferation [19], autoimmunity disease [20, 21], cardiovascular disease [22] and insulin synthesis [23]. Generally, the function of a microRNA is achieved via binding to the 3 prime untranslated region (3′-UTR) of target mRNA of a gene [24], with the result that the microRNA represses protein production [25]. Therefore, we used the bioinformatics tools to predict and analyze the target genes of gga-miR-15a, and twelve target genes of gga-miR-15a were significantly enriched in the insulin-signaling pathway. In the chicken liver, the role of the insulin-signaling pathway is similar to that in mammals, which has anabolic effects in glucose transport and utilization, glycogen synthesis, control of liver lipogenic enzymes, amino acid transport and protein synthesis [26]. Sun et al. [23] previously demonstrated that microRNA-15a positively regulated insulin synthesis by targeting uncoupling protein-2 (UCP-2) in mice. In this study, based on the molecular interaction analysis, *FOXO1* was identified as the most reliable target gene of gga-mir-15a among 12 target genes. *FOXO1*, a member of the forkhead box transcription factor class O (FOXO) family, is a direct transcriptional regulator of gluconeogenesis and glycolysis, reciprocally regulated by insulin, and has profound effects on hepatic lipid metabolism [27]. With the activation of *FOXO1*, gluconeogenic gene activity is upregulated, promoting glucose production in the liver and accounting largely for the hyperglycemia observed in diabetic individuals [28]. Based on this combined information, we inferred that gga-mir-15a could target *FOXO1* by binding to the 3′-UTR of *FOXO1* mRNA and then inhibit the protein expression of *FOXO1* involved in the insulin-signaling pathway, resulting in the alteration of FCR in chickens.

In the present study, only 5 and 2 hits were identified for FI and RFI at a suggestively significant level, respectively, which indicated that the effect of genetic determinants was too weak to be identified for FI and RFI. Additionally, a pure line selection scheme resulted in effective variants homozygous in the current stocks. However, the suggestively associated SNPs were also promising candidates to some extent. The identified SNPs on chromosome 9 (GGA9) for FI were first reported in chickens according to the QTL database (http://www.animalgenome.org/cgi-bin/QTLdb/GG/index). SNP *rs313839239* for RFI was overlapped with a QTL previously identified in commercial meat-type chickens [29]. Notably, because of the high genetic correlation between the two traits, we detected a consensus association (i.e., *rs313839239*) affecting RFI and FI simultaneously, which is similar to our findings in the laying period from 57 to 60 weeks in a previous study [5]. *FARP2* (FERM, RhoGEF and pleckstrin domain-containing protein 2), a guanine nucleotide exchange factor in the Rho family of small GTPases, was a shared candidate gene for both FI and RFI. *FARP2* was identified as a candidate gene in diabetes research [30], and is correlated with energy metabolism and obesity-associated pathologies [31]. Another promising candidate gene for RFI was cannabinoid receptor 1 (*CNR1*), which is referred to in an energy homeostasis and metabolic process revealed by pharmacological studies [32].

## Conclusions

In conclusion, the screened genomic region/variants for FCR, FI and RFI can be valuable references for designing the customized genetic and genomic selection schemes to improve efficiency of feed utilization in the current nucleus of breeding flocks. *MIR15A* should be considered a primary candidate gene to improve the understanding of the genetic and physiological factors affecting the FCR in investigated populations. The interactions between *MIR15A* and target genes, such as *FOXO1*, suggested that the insulin-signaling pathway in the liver might be the causative factor affecting FCR regulation in chickens. The mechanisms by which these relevant factors modulate metabolism and homeostasis in the pathway should be studied further in chickens. This new insight provided by our study will help to understand the biological regulation of metabolism and homeostasis in chickens.

## Materials and methods

### Animals and phenotypes

Female chickens from the 11th generation of a pedigreed line of Rhode Island Red were maintained by Beijing Huadu Yukou Poultry Breeding Co. Ltd., China with selection on egg production and quality. Birds were generated from the same hatch and housed in identically individual cages with free access to feed and water. Feed intake was individually collected during a 2-wk. (81–82 weeks of age) trial period. In the 2-wk. feeding test period, feed was provided in individual containers for each hen. Feed consumption data were procured daily by manual collection to derive the daily feed intake (FI). Egg mass (EM) was obtained as total egg number multiplied by average egg weight for each hen. Daily egg number was recorded to calculate total egg number, and the weight of 3 consecutive eggs collected in each week was used to calculate average egg weight. Body weight was measured on a mid-test day for each recorded hen. Feed conversion ratio (FCR) was calculated as the ratio of FI and daily EM. After removing outliers (values greater than 3 SD from the mean) for FCR, the remaining 808 hens were used to calculate residual feed intake (RFI) as the residuals from a regression model of FI on EM and metabolic BW (BW raised to the power of 0.75) [33]. The phenotypes that did not follow a normal distribution were transformed by Johnson method implemented in R software, and then transformed data were used in the next genetics analysis.

### Genotyping, imputation and quality control

Genomic DNA was isolated from whole blood samples using phenol-chloroform methods. The qualified 808 hens were genotyped for 580,961 markers using Affymetrix 600 K chicken high-density genotyping array. In the quality control of raw data, all individuals passed the criteria with a missing SNP call rate < 5% using Affymetrix power tool (APT) provided by Affymetrix (http://www.affymetrix.com/). Autosomal SNPs of 808 samples were filtered by the criteria set in PLINK [34] (sample call rate > 97%, minor allele frequencies >1% and Hardy Weinberg equilibrium *P*-value <1e-6). Afterward, the remaining SNPs and 808 birds were used for the imputation implemented in the Beagle Version 4 software package based on localized haplotype clustering [35]. Finally, a total of 307,216 SNPs distributed on 28 autosomes and 2 linkage groups, listed in Tables 5, and 808 birds were obtained for subsequent genetic analyses after filtering for imputation results using PLINK.

**Table 5:** Basic information for SNP markers on a physical map after quality control

Chromosome	Map distance (Kb)^a^	No. SNPs	Density (kb/SNP)	Chromosome	Map distance (Kb)	No. SNPs	Density (kb/SNP)
1	195,241.9	58,459	3.3	16	494.8	266	1.9
2	148,556.6	35,247	4.2	17	10,279.4	4954	2.1
3	110,445.2	33,201	3.3	18	11,198.7	5560	2.0
4	90,168.3	26,060	3.5	19	9979.3	4911	2.0
5	59,540.0	17,745	3.4	20	14,252.9	4843	2.9
6	34,904.9	11,610	3.0	21	6786.7	4808	1.4
7	36,195.7	12,537	2.9	22	4050.3	2234	1.8
8	28,724.0	9276	3.1	23	5700.7	3508	1.6
9	23,424.5	11,201	2.1	24	6313.8	4505	1.4
10	19,856.0	9704	2.0	25	2188.5	1494	1.5
11	19,381.0	7563	2.6	26	5288.3	3465	1.5
12	19,844.9	7772	2.6	27	5143.2	2922	1.8
13	17,425.5	6088	2.9	28	4735.4	2845	1.7
14	15,145.4	8102	1.9	LGE64	953.8	94	10.1
15	12,624.9	6203	2.0	LGE22^b^	739.1	39	19.0
Total	919,583.7	307,216					

^a^The physical length of the chromosome was based on the position of the last marker in the *Gullus gullus* version 4

^b^LGE22, linkage group LGE22C19W28_E50C23

### Evaluation of genetic parameters

Prior to the genetic analysis, the effect cage tiers were tested using analysis of variance implemented in SAS software, and effect of cage tiers was excluded from the next analysis due to lack of the significance. Pedigree-based genetic parameters for FI, RFI and FCR were estimated with the average information restricted maximum likelihood (AI-REML) method implemented in DMU software [36]. The multi-trait animal model adopted in the current analysis was the following:

$$ \mathrm{y}=1\upmu +\mathrm{Za}+\mathrm{e} $$

where y is the phenotypic value of each trait; 1 and Z are the incidence matrix of fixed effects (population means) and random effects (individual additive genetic effect), respectively; μ is the vector of fixed effects of population means; and a and e are the random additive effects and residual effect, respectively. Estimation of the phenotypic variance explained by significantly associated SNPs and all SNPs (SNP-based heritability [37] and SNP-based genetic correlation [38]) was calculated by restricted maximum likelihood (REML) analysis implemented in GCTA software [39].

### Genome-wide association analysis

Before the genome-wide association study (GWAS), the eligible SNPs and birds were used to evaluate the population structure by PLINK. First, all SNPs were pruned to obtain independent SNP markers using the indep-pairwise option, with a window size of 25 SNPs, a step of 5 SNPs, and r^2^ threshold of 0.2. Second, pairwise identity-by-state (IBS) distances were calculated between all individuals using the independent SNP markers. Finally, we calculated multidimensional scaling (MDS) components using the mds-plot option based on the IBS matrix, which was included as a covariate in the subsequent association analyses. GWAS was performed using a mixed models approach [40] implemented in the GEMMA software package, which fitted a linear mixed model to account for population stratification and sample structure with a faster computational time [41]. Association test with univariate linear mixed model (univariate GWAS) was performed for each trait. The statistical model was the following:

$$ \mathrm{y}=\mathrm{W}\upalpha +\mathrm{x}\upbeta +\mathrm{u}+\upvarepsilon $$

where y is the vector of traits value for all individuals; W is a matrix of covariates (fixed effects contain first 4 MDS components and a column of 1 s); α is a vector of the corresponding coefficients including the intercept; x is a vector of marker genotypes; β is the effect size of the marker; u is a vector of individual random effects; ε is vector of errors. The Wald test statistic *P*-value was used as the criterion to screen SNPs associated with the investigated traits. Conditional GWA analyses were performed using the same mixed model with the addition of the dosage of the strongest associated SNP as a covariate [42].

### Statistical and bioinformatics analyses

With respect to the *P*-value threshold of genome-wide significance, the simpleM method [43] was used to infer the independent tests. A total of 38,715 independent tests over the entire autosomal SNPs were obtained, and then genome-wide significance and suggestive significance were calculated as 1.29e-6 (0.05/38,715) and 2.58e-5 (1.00/38,715), respectively. The Manhattan and Q-Q plots were constructed for each trait by the GAP package (http://cran.r-project.org/web/packages/gap/index.html) within the R software. Linkage disequilibrium (LD) analysis was performed for the chromosomal regions with many associated SNPs clustered implemented in Haploview version 4.2 [44] with the algorithm proposed by Gabriel et al. [45].

SNP positions and information were obtained using annotation of *Gallus gallus* 4.0 genome version, and genes within 500,000 base pairs flanking the associated SNPs were chosen for further analysis. Target genes of microRNA were predicted using TargetScan (http://www.targetscan.org) and miRDB (http://www.mirdb.org/miRDB/). Kyoto Encyclopedia of Genes and Genomes (KEGG) pathway analysis was used to analyze target genes of microRNA implemented online with the DAVID platform (https://david.ncifcrf.gov/). Bioinformatics analyses of molecular interactions between microRNA and predicted coding genes were performed using RNAhybrid online [46] in which minimal free energy (MFE) of interaction less than −20 was considered binding. The mature miRNA sequence was downloaded from miRBase (http://www.mirbase.org/), and the target sequence of coding genes was queried from Ensembl online (http://www.ensembl.org/).

### Total RNA extraction and qRT-PCR

Birds were deeply anesthetized with sodium pentobarbital via cardiopuncture, and decapitated. Liver tissue samples were collected from chickens along with the regular quarantine inspection of the experimental station of China Agricultural University in accordance with the Guidelines for the Care and Use of Experimental Animals established by the Ministry of Agriculture of China (Beijing, China). The entire study was approved by the Animal Welfare Committee of China Agricultural University (permit number: SYXK 2007–0023).

Total RNA was extracted from the liver tissue using a mirVana™ miRNA Isolation Kit (Life Technologies, Carlsbad, CA, USA), and was reversely transcribed using a miRACLE cDNA Synthesis Kit (Genetimes Technology, Shanghai, China) as described by the manufacturer. The quantitative real-time PCR (qRT-PCR) was performed on an ABI 7500 system (Applied Biosystems). Primers of gga-miR-15a and chicken 5 s rRNA were designed and synthesized by Genetimes Technology Inc. (Shanghai, China). The mature miRNAs were polyadenylated by polyA polymerase and reversely transcribed into complementary DNA (cDNA) using a reverse primer, which had a 3 prime degenerate anchor and a universal tag sequence on the 5 prime end. The cDNA template was then amplified using specific forward and universal reverse primers. The specific forward primer sequences of the miRNAs used in this study were the following: gga-miR-15a, forward 5′-TAGCAGCACATAATGGTTTGTAAAA-3′ and chicken 5 s rRNA, forward 5′-ACCGGGTGCTGTAGGCTTAA-3′. The universal reverse primer was included in the qPCR Kit of miRACLE qPCR miRNA Master Mix (Genetimes Technology). The optimum thermal cycling conditions were as follows: 95 °C for 10 min, 40 cycles of 95 °C for 10 s, 60 °C for 20 s, 72 °C for 1 min, 95 °C for 15 s, 60 °C for 30 s, and 95 °C for 15 s. All experiments were run in triplicate. Relative quantification of microRNA expression was analyzed using the ΔΔCT method [47] with 5 s RNA as the endogenous control and the average of the birds in HFCR group as the calibrator sample. Relative quantities calculated as 2^-ΔΔCT^ were used for statistical analyses. Data were analyzed by pairwise Student’s t-tests implemented in the R software.

## Additional files

Additional file 1: Table S1. The information for SNPs suggestively associated with feed conversion ratio. **Table S2.** Predicted target genes from TargetScan for gga-mir-15a. **Table S3.** Predicted target genes from miRDB for gga-mir-15a. (XLSX 37 kb)

Additional file 2: Figure S1. Linkage map of the associated genomic region for daily feed intake on chicken chromosome 9. (TIFF 166 kb)

Additional file 3: Figure S2. Regional association plot for FCR after conditional analysis on *rs13553102*. The graph plots genomic position (x axis) against its significance expressed as -log10 *P* value (y axis). (TIFF 205 kb)

Additional file 4: Figure S3. Molecular interactions between gga-mir-15a and target genes with minimal free energy less than −20 kcal/mol. Red letters indicate the 3′ UTR sequences of the target genes. Green letters indicate the matured sequences of gga-miR-15a. (TIFF 3113 kb)

## Abbreviations

AI-REML: Average information restricted maximum likelihood
DAVID: Database for annotation, visualization and integrated discovery
EM: Daily egg mass
FCR: Feed conversion ratio
FI: Daily feed intake
GGA: *Gallus gallus* chromosome
GIF: Genomic inflation factors
GWAS: Genome-wide association study
KEGG: Kyoto Encyclopedia of Genes and Genomes
LD: Linkage disequilibrium
MAF: Minor allele frequency
MBW: Mean body weight
MFE: Minor free energy
qRT-PCR: Quantitative real time polymerase chain reaction
RFI: Residual feed intake
SNP: Single nucleotide polymorphism
