# Supplementary figures and images for: Genome-wide association study reveals putative role of gga-miR-15a in controlling feed conversion ratio in layer chickens
Source: BMC Genomics. 2017 Sep 6;18:699. doi: 10.1186/s12864-017-4092-9 (PMC5586008; doi:10.1186/s12864-017-4092-9)

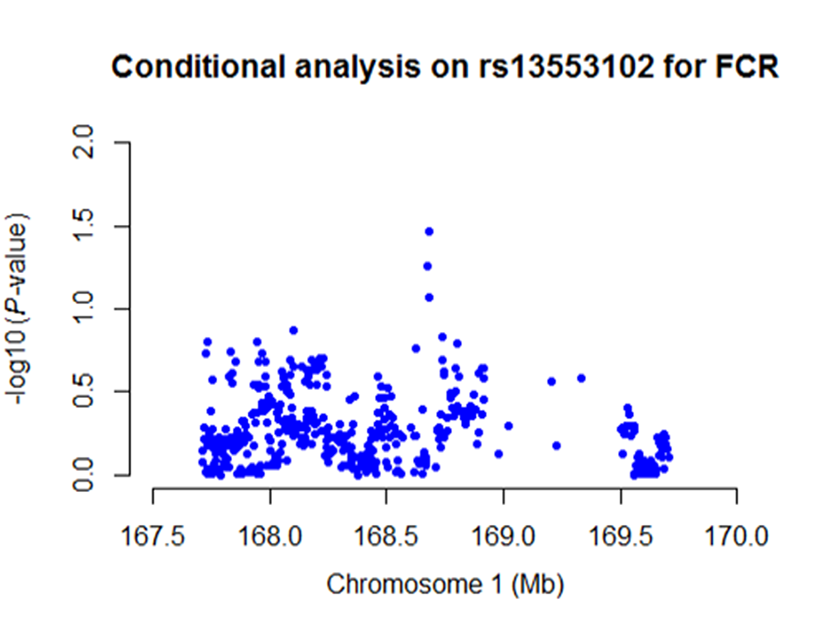

Supplement: Supplementary file 2 — Linkage map of the associated genomic region for daily feed intake on chicken chromosome 9. (TIFF 166 kb) [file 12864_2017_4092_MOESM2_ESM.tif]

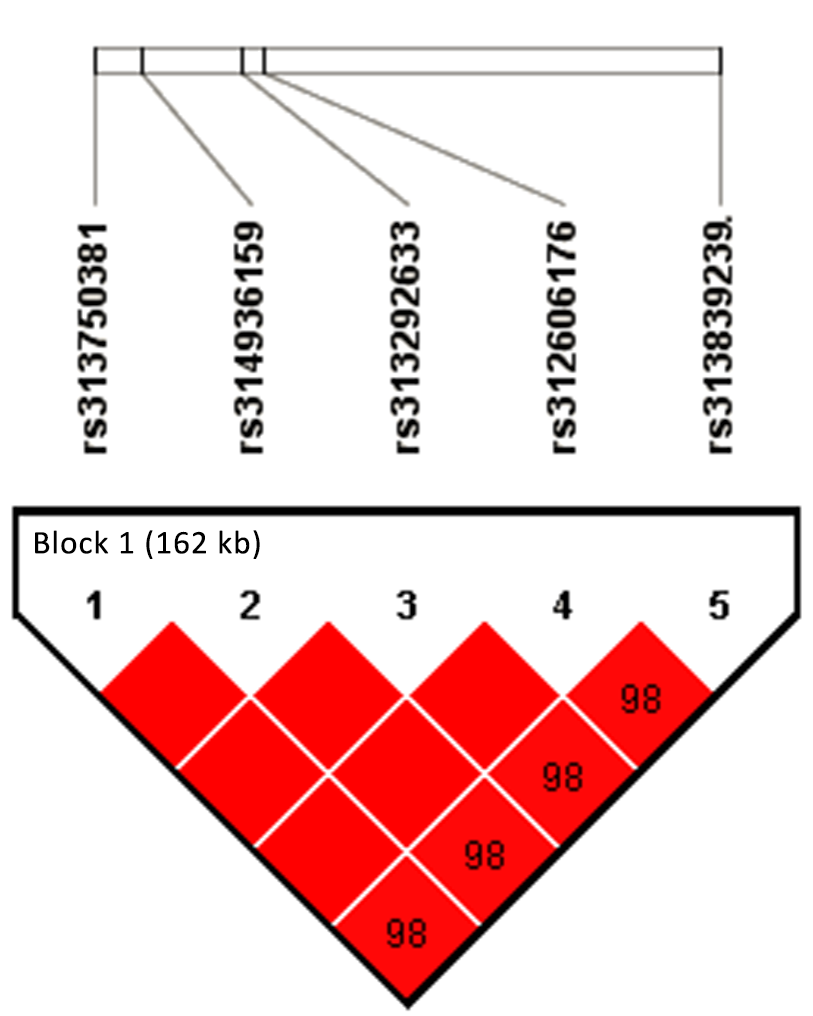

Supplement: Supplementary file 3 — Regional association plot for FCR after conditional analysis on rs13553102. The graph plots genomic position (x axis) against its significance expressed as -log10 P value (y axis). (TIFF 205 kb) [file 12864_2017_4092_MOESM3_ESM.tif]

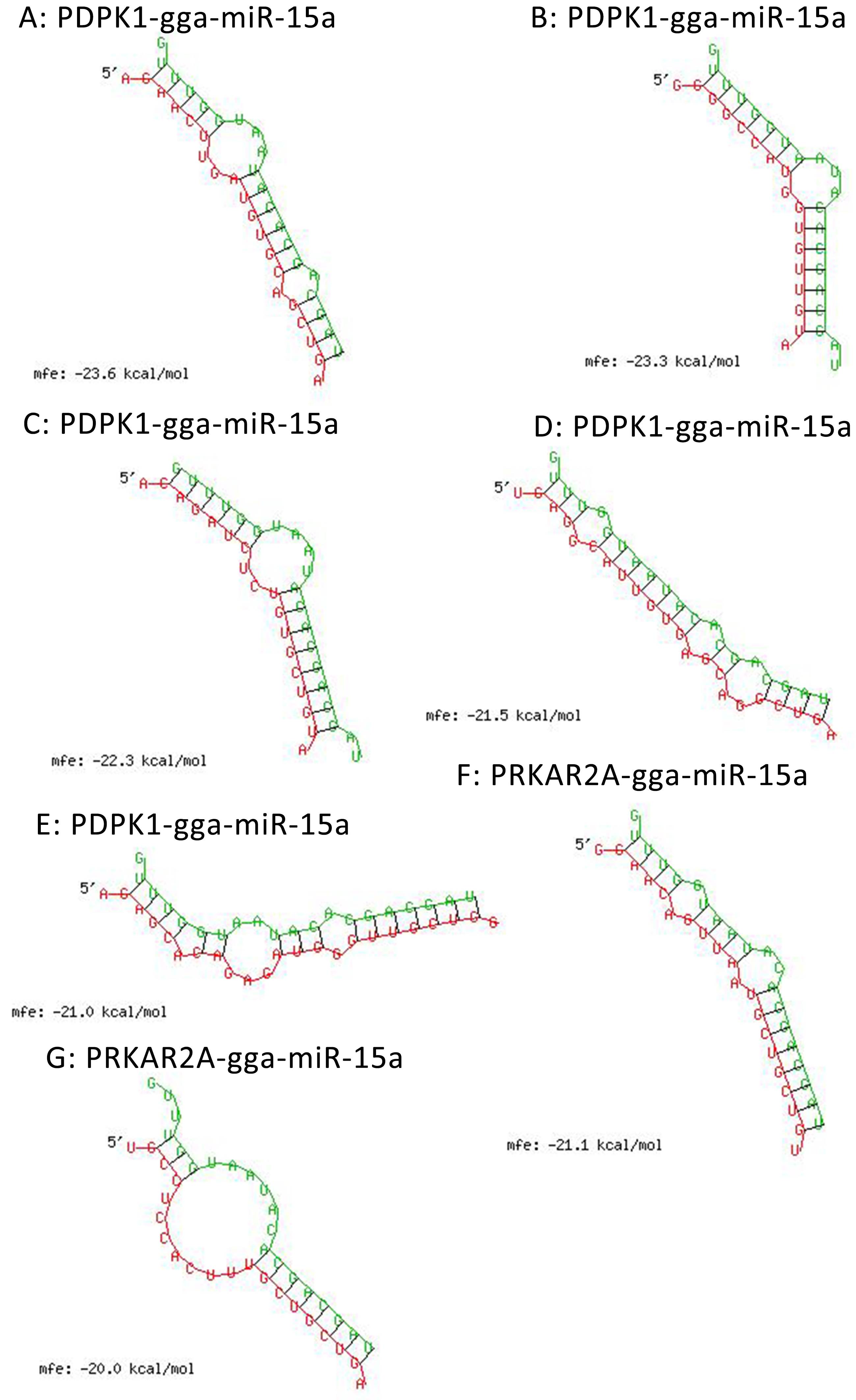

Supplement: Supplementary file 4 — Molecular interactions between gga-mir-15a and target genes with minimal free energy less than −20 kcal/mol. Red letters indicate the 3′ UTR sequences of the target genes. Green letters indicate the matured sequences of gga-miR-15a. (TIFF 3113 kb) [file 12864_2017_4092_MOESM4_ESM.tif]
